# Comparing intra-uterine injection of mononuclear cells and platelet-rich plasma on the pregnancy rate of women with recurrent implantation failure: An RCT

**DOI:** 10.18502/ijrm.v22i10.17668

**Published:** 2024-12-02

**Authors:** Hoda Fazaeli, Azar Sheikholeslami, Zahra Ebrahimi, Naser Kalhor, Leila Naserpour

**Affiliations:** ^1^Department of Cell Biology and Regenerative Medicine, Academic Center for Education, Culture, and Research (ACECR), Qom Branch, Qom, Iran.; ^2^Department of Reproductive Biology, Academic Center for Education, Culture, and Research (ACECR), Qom Branch, Qom, Iran.

**Keywords:** Peripheral blood mononuclear cells, Platelet-rich plasma, Recurrent implantation failure, Pregnancy.

## Abstract

**Background:**

Recurrent implantation failure (RIF) can be explained mainly by improper crosstalk between the embryo and endometrium. The T-helper1/T-helper2 profile balance influences effective embryo implantation. Endometrial immunomodulation via intrauterine injection of activated peripheral blood mononuclear cells (PBMCs) or autologous platelet-rich plasma (PRP) is a potentially efficient treatment option.

**Objective:**

This study aims to examine the biochemical and clinical pregnancies resulting from the intrauterine administering of activated PBMCs and PRP in RIF women.

**Materials and Methods:**

This randomized clinical trial study was done in the Rooya Infertility Treatment Center, Qom, Iran from November 2022 to April 2024. 96 women with at least 2 RIFs were randomized into control, PBMC, and PRP groups. Briefly, 3 ml of blood sample was collected and PBMCs were isolated using Ficoll separation solution, and cultured for 72 hr. PRP was separated from 10 ml of peripheral blood through centrifugation. 2 days before embryo transfer PBMCs or PRP were transferred into the endometrial cavity.

**Results:**

Except for the duration of infertility, which was higher in the PBMC group, all other baseline characteristics were not statistically different. Moreover, a significantly higher rate of biochemical pregnancy was observed in the PRP (10/32) and PBMC (12/32) groups compared to the control (3/32) (p = 0.027), while the rate of clinical pregnancy was only significantly higher in the PBMC group (10/32) than in the control group (2/32) (p = 0.038).

**Conclusion:**

Neither PBMC nor PRP interventions exhibited a substantial advantage over one another regarding biochemical and clinical pregnancy rates.

## 1. Introduction

In vitro fertilization (IVF) success rates in assisted reproductive technology (ART) heavily rely on embryo implantation, which only happens approximately in 20–30% of women who have had a fresh embryo transfer (ET). However, following several IVF procedures, about 10% of cases experience at least 2 or 3 unexplained repeated implantation failures (RIFs) (1).

RIF has been associated with a variety of reasons, the most common of which are multifactorial, endometrial, and idiopathic. Infections, lifestyle modifications, gamete and embryo quality, maternal and paternal variables, psychological conditions, and oxidative stress can all contribute to multifactorial RIFs (2, 3). Endometrial RIF can be explained by impaired endometrium function, such as aberrant growth or loss of vascularization; however, the main question unanswered and need for research surrounding idiopathic RIF is mostly brought on by improper crosstalk between the embryo and endometrium (2). A lack of early inflammation may be the primary factor causing implantation failure in RIF women. While immune cells including T lymphocytes and macrophages, which are crucial for initiating inflammation are recruited at the maternal-fetal interface after successful implantation. They help the endometrium and blastocyst to produce more cytokines, resulting in a profile dominated by T-helper1 (Th1) pro-inflammatory cytokines (4, 5). However, activation of a specific cytokine profile is required, which is characterized by a high level of T-helper2 (Th2) cytokines and causes an anti-inflammatory state appropriate for uterine receptivity, subsequently leading to pregnancy (6–8). Thus, effective embryo implantation is influenced by the Th1/Th2 profile balance, which is regulated by T lymphocytes and uterine natural killers (4, 9, 10).

A potential treatment option is endometrial immunomodulation via intrauterine injection of activated peripheral blood mononuclear cells (PBMCs) or autologous platelet-rich plasma (PRP). By controlling the Th1/Th2 cytokine ratio and growth factors to trigger several cytokine cascades and matrix metalloproteinase actions, intrauterine treatment of PBMCs in women with RIF seeks to increase endometrial receptivity (2, 4, 5). It has been established that an elevated Th1/Th2 ratio in peripheral blood is harmful for embryo implantation. However, PBMCs generate a large number of cytokines that can control the Th1/Th2 imbalance in RIF-affected women (11). At the point of embryo implantation, PBMCs are also known to stimulate the secretion of growth factors as well as Th1 proinflammatory and anti-inflammatory cytokines, which improve endometrial receptivity (12, 13). On the other hand, it has been observed that autologous PRP increases endometrial growth through activating cytokines and growth factors such as transforming growth factor, platelet-derived growth factor, epidermal growth factor, and vascular endothelial growth factor leading to improved pregnancy outcomes during IVF (14). These growth factors can control cell migration, attachment, proliferation, and differentiation while promoting the accumulation of extracellular matrix (15).

The aim was to examine the biochemical and clinical pregnancies resulting from intrauterine administering activated PBMCs and PRP in RIF women.

## 2. Materials and Methods

This randomized clinical trial was done in the Rooya Infertility Treatment Center of the Academic Center for Education, Culture, and Research, Qom, Iran, from November 2022 to April 2024. 96 women with at least 2 RIF following IVF/intracytoplasmic sperm injection (ICSI) were randomized into control, PBMC, and PRP groups. Participants with at least 2 RIF in IVF/ICSI, primary infertility, endometrial thickness < 6 mm at the time of ovulation induction, < 40 yr, regular menstrual cycles, body mass index of < 30, absence of uterine pathology and infections, unexplained infertility, and infertility with tubal causes were included in the study, while polycystic ovarian syndrome, uterine pathology, male factor infertility, presence of hydrosalpinx, and endometriosis were considered as the exclusion criteria.

The selected participants, women who signed written informed consent, were allocated to receive autologous PBMC cultures or PRP via noninvasive intrauterine insemination 2 days before ET, representing the PBMC and PRP groups, respectively, while receiving no treatment at all before ET acted as the control group.

### Sample size

A study in Japan (5) injected PBMCs into the uterus of people with RIF, where the pregnancy rate was equal to p = 31 in the intervention group and was equal to p_2_ = 5.5 in the control group, indicating that a minimum sample size of 32 cases will be needed for this study in each group. This is based on the study's 80% power and 5% type 1 error. This way, a total of 96 women were studied. Using MedCal statistical software and the following formula, sample size calculations were performed. 


$$n=\frac{2\left(\bar{p}\right)\left(1-\bar{p}\right){\left(Z_{\beta }+Z_{\alpha /2}\right)}^{2}}{{\left(P_{1}-p_{2}\right)}^{2}}$$

### Randomization and blinding

Participants were randomly allocated into 3 groups, control, PBMC, and PRP. Based on the epidemiologist's suggestion, a package containing 96 colored cards, 32 of each color, white, blue, and red were used for randomization. After checking the participants' eligibility by the specialist, the researcher divided them into groups by randomly selecting one of the colored cards from the box (without revealing the contents). In this way, the white color represented the control group, blue represented the PBMC group, and red for the PRP group. The participants were not informed about the type of treatment they had received to maintain study blindness. Additionally, because the doctors who performed the PBMC or PRP therapy, the induction cycle, and the ET were not the same, the ET was carried out without the participant's treatment history being known. So that their ET procedure was unaffected by their knowledge of this issue, it should be highlighted that the statistical analysis of the collected data was performed by a different researcher who was not aware of the treatments received by the various groups.

### Stimulation protocol

The antagonist protocol employing (Cetrotide 0.25, Gonal-F) recombinant follicle-stimulating hormone (Cinnal-f, Iran) was preferred to reduce the impact of the treatment and improve sample coordination. Subsequently, daily subcutaneous injections of recombinant follicle-stimulating hormone (150–225 IU/day) were started and continued for 5 days, while being adjusted by standard follicle growth criteria measured by blood estradiol concentrations and ultrasound monitoring. Ultrasound scans were used to determine the number and size of primary antral follicles as well as the average ovarian volume.

The 3 ${\;}^{\text{rd}}$ -generation gonadotropin-releasing hormone antagonist cetrorelix acetate (cetrotide, 3 mg) was administered once subcutaneously, at least 1 follicle with a diameter of 14 ≤ mm was observed, and patients were seen 3 days later for the next monitoring visit. After the follicles reached a size of ≥ 17 mm, an intramuscular injection of 10,000 IU of human chorionic gonadotropin (Gonarex, Iran) was administered. 50 mg progesterone ampoule (Iran Hormone, Iran) was used to induce the luteal phase from the day of egg collection to the day of the pregnancy test. In the case of pregnancy, progesterone continued until the fetus's heart activity was detected.

### Preparing autologous PBMCs and PRP

#### Isolation and culture of PBMCs

On the day of ovulation induction, a 3 ml blood sample was collected from RIF women undergoing ART cycles and placed in citrated tubes to perform a separation based on the Ficoll-Hypaque solution (Biowest, France). We found a separate layer of PBMC under the median plasma after centrifugation at 18–20 C for 30–40 min at 400 g. To obtain a PBMC pellet, the PBMC milky layer was transferred to another tube and washed twice with phosphate-buffered saline through centrifugation at 60–100 g for 10 min. The pellet was then resuspended in Roswell Park Memorial Institute Culture Media (Biowest, France) with 75 IU of human menopausal gonadotropin (Menotropin, Iran) at 37 C. 2 days before ET which coincided with 72 hr of incubation of PBMCs resulted in the development of cell clusters, 1–1.5 × 10^6^ cells in 0.5 ml medium were transferred into the endometrial cavity.

#### Preparation of PRP

A commercial kit (Rooyagen, Iran) was used to prepare PRP. 2 days before ET, about 10 ml of peripheral venous blood was drawn into a syringe containing 2.5 ml of citric acid anticoagulant solution and immediately centrifuged for 10–12 min at 1700 rpm to separate the red blood cells. Then the plasma was centrifuged again at 3800 rpm for 7 min to obtain PRP, which contains 4–5 times more platelets than peripheral blood. Around, 0.5–1 ml of the resulting PRP was injected into the uterine cavity with a transfer catheter (Idea Varzan Farda, Iran), 2 days before ET.

### ET

Following the ICSI or IVF procedure, the 2 best day 3 embryos were transferred into the uterus of the women receiving PBMC, PRP, or no treatment. Embryos were maintained in culture until day 3 after ICSI or IVF and their quality was assessed. An embryo was considered to be of adequate quality if its blastomeres were of consistent size and shape, while acceptable quality embryos were those whose ooplasm had a maximum fragmentation of 10% and no granularity (A + B). Based on the participants condition, 1–3 embryos were transplanted in utero using a Frydman catheter (CCD Laboratories, Paris, France).

### Variable assessments

The age of participants and their partners, duration of infertility, the number of RIFs, and the number of total and good-quality embryos transferred per case were assessed statistically among groups. In addition, biochemical and clinical pregnancy rates as primary outcomes were assessed: 2 wk after gestation a beta-human chorionic gonadotropin ( β -hCG) blood test value of > 20 IU/L was considered as biochemical pregnancy, while clinical pregnancy was controlled by using ultrasound at 3–5 wk of gestation to check for fetal viability.

### Ethical Considerations

Each participant received information about the objectives of the study and was asked to sign a consent form. The Declaration of Helsinki was followed in the conduct of this investigation. The prospective randomized double-blinded clinical trial was approved by the Ethics Committee of the Iranian Academic Center for Education, Culture, and Research, Qom, Iran (Code: IR.ACECR.REC.1398.002) and was registered in the Iranian registry for clinical trials, which was updated on July 11, 2024.

### Statistical Analysis

The data analysis was conducted utilizing IBM Corp Statistical Package for the Social Sciences (SPSS) version 22.0. For normally distributed data, the continuous variables are shown as means ± standard deviations, and the one-way ANOVA test was applied to compare the variations in participants features. A post hoc analysis for multiple comparisons was carried out using Tukey's test. The Chi-square test was used to compare the categorical variables, displayed as frequencies (percentages). At p < 0.05, statistical significance was established.

## 3. Results

**Figure 1:**
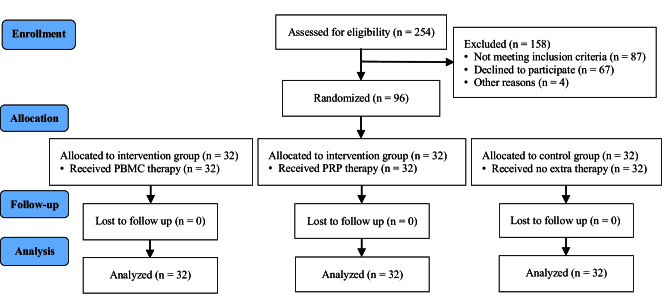
CONSORT flowchart.

Among the 254 patients who were assessed for eligibility, 87 were excluded from the study due to not meeting the intervention criteria, 67 were unwilling to participate, and 4 were excluded for other reasons. Finally, 96 eligible women were randomized into 3 groups of control, PBMC, and PRP (32 in each group). Fortunately, none of the participants were excluded or lost to follow-up after randomization (Figure 1). The comparison of clinical variables between the groups is shown in table I. While the age of participants and their partners, number of RIFs, number of total and good-quality embryos transferred per participant, and endometrial thickness were not statistically different among the groups, the duration of infertility was significantly different (p = 0.035) (Table I), whereas subsequential analysis showed longer infertility duration in PBMC group comparing to the control participants (p = 0.044).

Moreover, according to table II, no significant differences were observed in the serum level of follicle-stimulating hormone, anti-Müllerian hormone, and estradiol hormone among the control, PBMC, and PRP groups.

To evaluate the effect of PBMC and PRP treatments on the reproductive outcomes of women with RIF, we calculated the biochemical and clinical pregnancy rates in 3 groups (Table III). The percentage of biochemical and clinical pregnancies were significantly different among groups (p = 0.027 and p = 0.038, respectively). Therefore, a subsequent statistical test (Chi-square test) was done, which showed significantly higher biochemical pregnancy in the PBMC and PRP groups compared to the control group (p = 0.016 and p = 0.03, respectively). Additionally, the clinical pregnancy rate was not significantly different between PRP and PBMC or control groups (p = 0.396 and p = 0.302, respectively), while in comparison to the control group, the rate of clinical pregnancy was significantly higher (p = 0.03). Moreover, according to table IV the biochemical pregnancy rate in PBMC and PRP groups was respectively 5.8 and 4.394-fold more than the control group, while in case of clinical pregnancy rate, it was 6.818 and 4.091-fold higher than control group.

**Table 1:** Baseline characteristics in the control, PBMC, and PRP groups

	**Groups**			
**Parameter**	**Control**	**PBMC**	**PRP**	**P-value**
	**Female age (yr)**	32.94 ± 4.196 (33, 7)	32.72 ± 4.168 (33, 6)		31.41 ± 5.198 (30, 6.75)	0.350
**Male age (yr)**	35.37 ± 4.7 (36, 6.75)	35.41 ± 4.17 (35, 6)	34.03 ± 5.04 (34, 7.75)	0.406
**Duration of infertility (yr)**	7.84 ± 2.371 (8, 3)	9.3 ± 2.478 (9.25, 4.75)	8.04 ± 2.315 (7.25, 3.75)	0.035
**Number of RIFs**	2.56 ± 0.715 (2, 1)	2.78 ± 0.706 (3, 1)	2.68 ± 0.737 (3, 1)	0.66
**Number of embryos transferred per** **participant**	2.53 ± 0.841 (3, 1)	2.84 ± 0.919 (3, 1)	2.34 ± 0.865 (2, 1)	0.406
**Number of good-quality embryos (A + B)** **transferred per participant**	16 ± 9.899 (18, 8)	16 ± 4.242 (18, 5)	15 ± 4.242 (17, 5)	0.958
**Endometrial thickness (mm)**	10.56 ± 1.189 (10.25, 2.5)	10.94 ± 0.948 (11, 1.875)	10.61 ± 1.204 (11, 2.375)	0.345
Data presented as Mean ± SD (median, interquartile range), One-way ANOVA test. PBMC: Peripheral blood mononuclear cells, PRP: Platelet-rich plasma, RIFs: Repeated implantation failures				

**Table 2:** Comparison of hormonal levels in the control, PBMC, and PRP groups

	**Groups**			
**Hormone**	**Control**	**PBMC**	**PRP**	**P-value**
	**FSH**	5.87 ± 2.302 (5.6, 3.2)	5.75 ± 1.516 (5.15, 3.225)		5.31 ± 2.123 (5.8, 2.125)	0.508
**AMH**	4.36 ± 2.53 (3.97, 4.325)	4.6 ± 2.603 (4.3, 3.2)	3.55 ± 1.414 (3.59, 2.19)	0.158
**Estradiol**	41.22 ± 8.62 (38.5, 7.25)	40.54 ± 10.51 (39.5, 13)	44.72 ± 9.89 (47, 16.75)	0.186
Data presented as Mean ± SD (median, interquartile range), One-way ANOVA test. PBMC: Peripheral blood mononuclear cells, PRP: Platelet-rich plasma, FSH: Follicle-stimulating hormone, AMH: Anti-Müllerian hormone				

**Table 3:** Comparison of pregnancy rates in the control, PBMC, and PRP groups

	**Groups**			
**Pregnancy**	**Control**	**PBMC**	**PRP**	**P-value**
	**Biochemical**	3 (9.37)	12 (33.5)		10 (31.25)	0.027
**Clinical**	2 (6.25)	10 (31.25)	6 (18.75)	0.038
Data presented as n (%), Chi-square test. PBMC: Peripheral blood mononuclear cells, PRP: Platelet-rich plasma				

**Table 4:** The odd ratio (95% confidence interval) of pregnancy rate

[2.5in,lr]**Groups** **Pregnancy**	**Biochemical**	**Clinical**
**Control**	1	1
**PBMC**	5.8 (1.448–23.228)	6.818 (1.1356–34.274)
**PRP**	4.394 (1.079–17.893)	4.091 (0.753–22.22)
PBMC: Peripheral blood mononuclear cells, PRP: Platelet-rich plasma		

## 4. Discussion

To our knowledge, the effects of PRP or PBMC intrauterine perfusion before ET on the pregnancy outcomes of RIF women have never been compared. Based on our obtained data, except the duration of infertility which was significantly higher in the PBMC group than the control, all other baseline characteristics including assessed hormonal level, age of partners, number of RIFs, and endometrial thickness were not statistically different, which means our study population was homogenously recruited. Moreover, our study demonstrated that there was a significantly higher rate of biochemical pregnancy (positive β -hCG) following the intrauterine administration of PRP or PBMC compared to the control group, while the rate of clinical pregnancy (observation of gestational sac) was only significantly higher in the PBMC group than the control group.

Over the past 17 yr, several research have been done on the use of PBMCs for endometrial immunomodulation in RIF women (4, 16–18). In some infertile women with unexplained pathology who are candidates for intrauterine insemination, the endometrial response to ovarian stimulation may not be identified as a clinical problem until they have numerous treatment failures (17). This condition may be linked to a pro-inflammatory state, which is necessary for early embryo implantation. To ensure implantation and the progression of gestation, the anti-inflammatory profile should be given prominence (19). It is reported that PBMC treatment leads to the production of more anti-inflammatory cells after 48 hr of culture (17). In addition, by promoting endometrial differentiation, PBMCs can aid in embryo adhesion and invasion (20). PBMCs generate a guided pathway for attachment and invasion of the embryo through its movement toward the stromal tissue of the endometrium. Surface molecules on endometrial epithelial cells are changed in form and function by the protease produced by PBMCs (21). This explains why women with RIF benefit from intrauterine administration of PBMCs.

There are divergent opinions about PRP perfusion therapy's efficacy in IVF reproductive medicine. For example, in research that was in line with our findings in the case of the rate of positive β -hCG in PRP therapy-receiving RIF women, the rate of live births and clinical pregnancies were significantly higher in these individuals which is in contrast with our results. However, they reported that the rates of implantations and miscarriages did not differ significantly in comparison to control RIF women (22). Furthermore, autologous PRP therapy has been shown to expand the thin endometrium and raise clinical pregnancy and implantation rates in frozen ET cycles (23–25). It is believed that PRP may regenerate and enhance endometrial receptivity, resulting in successful pregnancy outcomes (26). In contrast, some reports have shown that PRP intrauterine infusion in RIF women undergoing frozen-thawed ET cycles causes improved clinical and continuing pregnancy and live birth rates compared to the control group (27, 28). The discrepancy between our findings and those of other researchers may be ascribed to the various subject cohorts, variations in RIF definitions, and multiple PRP preparation techniques.

There is a comprehensive agreement about the effectiveness of PBMCs intrauterine delivery for RIF women through raising implantation and pregnancy rates in women who have had 3 or more prior implantation failures (29, 30). For example, a study has confirmed the effectiveness of PBMC that was stimulated in vitro with corticotropin-releasing hormone (CRH) for 48–72 hr in women undergoing frozen-thawed ET who had 3 or more implantation failures (1). Another in vitro study applied activated PBMCs with 10 IU/mL hCG for 48 hr before the intrauterine treatment, specifically in utero of RIF women with reduced Th-17/treg cell ratios, leading to dramatically improved clinical pregnancy and live birth rates and decreased miscarriage rates. Additionally, they demonstrated that the PBMCs culture media had higher concentrations of 3 pro-inflammatory cytokines, interleukin-1-beta, tumor necrosis factor-alpha, and interferon-gamma, indicating that PBMCs produce these Th1 cytokines in reaction to hCG treatment (31).

Furthermore, a research has demonstrated that autologous PBMCs treated with CRH before blastocyst or early, cleaved ET produced superior outcomes for clinical pregnancy in women with RIF than PBMCs without CRH treatment (32). Our findings in the case of PBMC therapy are in line with all the aforementioned studies, whereas human menopausal gonadotropin-activated PBMCs led to elevated rates of biochemical and clinical pregnancies.

## 5. Conclusion

In summary, although PBMC therapy as an immunomodulation strategy for RIF infertile individuals may be a suitable approach, neither PBMC nor PRP interventions exhibited a substantial advantage over one another in terms of both biochemical and clinical pregnancy rates. However, more studies with larger sample sizes are required to compare these 2 complementary treatments in ART.

##  Data Availability

The data that support the findings of this study are available on request from the corresponding author.

##  Author Contributions

Conceptualization: L. Naserpour, Z. Ebrahimi. Data acquisition: H. Fazaeli, A. Sheikholeslami, L. Naserpour. Data analysis or interpretation: N. Kalhor, L. Naserpour. Drafting of the manuscript: H. Fazaeli, A. Sheikholeslami. Critical revision: A. Sheikholeslami, H. Fazaeli.

##  Conflict of Interest

The authors declare that there is no conflict of interest.
